# Further research on complete moment convergence for moving average process of a class of random variables

**DOI:** 10.1186/s13660-017-1322-2

**Published:** 2017-02-17

**Authors:** Yong Zhang, Xue Ding

**Affiliations:** College of Mathematics, Jilin University, Changchun, 130012 P.R. China

**Keywords:** complete moment convergence, moving average process, Rosenthal type maximal inequality, slowly varying function

## Abstract

In this article, the complete moment convergence for the partial sum of moving average processes $\{X_{n}=\sum_{i=-\infty}^{\infty}a_{i}Y_{i+n},n\geq 1\}$ is established under some mild conditions, where $\{Y_{i},-\infty < i<\infty\}$ is a doubly infinite sequence of random variables satisfying the Rosenthal type maximal inequality and $\{a_{i},-\infty< i<\infty\}$ is an absolutely summable sequence of real numbers. These conclusions promote and improve the corresponding results given by Ko (J. Inequal. Appl. 2015:225, 2015).

## Introduction

We first introduce the definition of the Rosenthal type maximal inequality, which is one of the most interesting inequalities in probability theory and mathematical statistics. Suppose that $\{ Y_{n},n\geq1\}$ is a sequence of random variables satisfying $E\vert Y_{i}\vert ^{r}<\infty$ for $r\geq2$, then there exists a positive constant $C(r)$ depending only on *r* such that

1.1 $$\begin{aligned} E\max_{1\leq j\leq n}\Biggl\vert \sum _{k=1}^{j}(Y_{k}-EY_{k})\Biggr\vert ^{r}\leq C(r)\Biggl[\sum_{k=1}^{n}E \vert Y_{k}-EY_{k}\vert ^{r}+\Biggl(\sum _{k=1}^{n}E\vert Y_{k}-EY_{k} \vert ^{2}\Biggr)^{r/2}\Biggr]. \end{aligned}$$

Equation (1.1) can be satisfied by many dependent or mixing sequences. Peligrad [2], Zhou [3], Wang and Lu [4], Utev and Peligrad [5] established the above inequality for *ρ*-mixing sequence, *φ*-mixing sequence, $\rho^{-}$-mixing sequence, and *ρ̃*-mixing sequence, respectively. We also refer to Shao [6], Stoica [7], Shen [8], Yuan and An [9] for negatively associated sequence (NA), martingale difference sequence, extended negatively dependent sequence (END), and asymptotically almost negatively associated random sequence (AANA), respectively.

The following definitions will be useful in this paper. The first one can be found in Kuczmaszewska [10].

### Definition 1.1

A sequence $\{Y_{i},-\infty< i<\infty\}$ of random variables is said to satisfy a weak dominating condition with a dominating random variable *Y* if

$$\sum_{i=j+1}^{j+n}P\bigl\{ \vert Y_{i}\vert >x\bigr\} \leq C nP\bigl\{ \vert Y\vert >x\bigr\} ,\quad x \geq0, -\infty < j< \infty, n\geq1, $$

where *C* is a positive constant.

### Definition 1.2

A real valued function $l(x)$, positive and measurable on $[0,\infty)$, is said to be slowly varying at infinity if for each $\lambda>0$, $\lim_{x\to\infty}\frac{l(\lambda x)}{l(x)}=1$.

Throughout the paper, let $\{Y_{i},-\infty< i<\infty\}$ be a sequence of random variables with zero means and $\{a_{i},-\infty< i<\infty\}$ be a sequence of real numbers with $\sum_{i=-\infty}^{\infty} \vert a_{i}\vert <\infty$, and the moving average process $\{X_{n}, n\geq1\}$ is defined by $X_{n}=\sum_{i=-\infty}^{\infty}a_{i}Y_{i+n}$. The complete moment convergence of moving average process $\{X_{n},n\geq1\}$ has been widely investigated by many authors. We list some results as follows.

Li and Zhang [11] established the following complete moment convergence of moving average processes under NA assumptions.

### Theorem A

*Suppose that* $\{X_{n}=\sum_{i=-\infty}^{\infty} a_{i}\varepsilon_{i+n}, n\geq1\}$, *where* $\{a_{i},-\infty< i<\infty\}$ *is a sequence of real numbers with* $\sum_{i=-\infty}^{\infty} \vert a_{i}\vert <\infty$ *and* $\{\varepsilon_{i},-\infty< i<\infty\}$ *is a sequence of identically distributed NA random variables with* $E\varepsilon_{1}=0$, $E\varepsilon_{1}^{2}<\infty$. *Let* *h* *be a function slowly varying at infinity*, $1\leq q<2 $, $r>1+q/2$. *Then* $E\vert \varepsilon_{1}\vert ^{r}h(\vert \varepsilon_{1}\vert ^{q})<\infty$ *implies*

$$\begin{aligned} \sum_{n=1}^{\infty}n^{r/q-2-1/q}h(n) E\Biggl\{ \Biggl\vert \sum_{j=1}^{n}X_{j} \Biggr\vert -\varepsilon n^{1/q}\Biggr\} ^{+} < \infty \end{aligned}$$

*for all* $\varepsilon>0$.

Later on, the following complete moment convergence of moving average processes generated by *ρ*-mixing sequence was proved by Zhou and Lin [12].

### Theorem B

*Let* *h* *be a function slowly varying at infinity*, $p\geq1$, $p\alpha>1$, *and* $\alpha>1/2$. *Suppose that* $\{X_{n},n\geq1\}$ *is a moving average process based on a sequence* $\{ Y_{i},-\infty< i<\infty\}$ *of identically distributed* *ρ*-*mixing random variables*. *If* $EY_{1}=0$ *and* $E\vert Y_{1}\vert ^{p+\delta }h(\vert Y_{1}\vert ^{1/{\alpha}})<\infty$ *for some* $\delta>0$, *then for all* $\varepsilon>0$,

$$\begin{aligned} \sum_{n=1}^{\infty}n^{p\alpha-2-\alpha}h(n) E\Biggl\{ \max_{1\leq k\leq n} \Biggl\vert \sum_{j=1}^{k}X_{j} \Biggr\vert -\varepsilon n^{\alpha}\Biggr\} ^{+} < \infty. \end{aligned}$$

Recently, Ko [1] obtained the complete moment convergence of moving average processes generated by a class of random variable.

### Theorem C

*Let* *h* *be a function slowly varying at infinity*, $p\geq1$, $p\alpha>1$, *and* $\alpha>1/2$. *Assume that* $\{ a_{i},-\infty< i<\infty\}$ *is an absolutely summable sequence of real numbers and that* $\{ Y_{i},-\infty< i<\infty\}$ *is a sequence of mean zero random variables satisfying a weak mean dominating condition with a mean dominating random variable* *Y* *and* $E\vert Y\vert ^{p}h(\vert Y\vert ^{1/{\alpha}})<\infty$. *Suppose that* $\{X_{n},n\geq1\}$ *is a moving average process based on the sequence* $\{ Y_{i},-\infty< i<\infty\}$. *Assume that the Rosenthal type maximal inequality of* $Y_{xj}=-xI\{Y_{j}<-x\}+Y_{j}I\{\vert Y_{j}\vert \leq x\}+xI\{Y_{j}>x\}$ *holds for any* $q\geq2$ *and* $x> 0$. *Then*, *for all* $\varepsilon>0$,

$$\begin{aligned} \sum_{n=1}^{\infty}n^{p\alpha-2-\alpha}h(n) E\Biggl\{ \max_{1\leq k\leq n} \Biggl\vert \sum_{j=1}^{k}X_{j} \Biggr\vert -\varepsilon n^{\alpha}\Biggr\} ^{+} < \infty. \end{aligned}$$

The aim of this paper is to study the complete moment convergence of moving average process of random sequence under the assumption that the random variables satisfy the Rosenthal type maximal inequality and the weak mean dominating condition. The paper is organized as follows. In Section 2 we describe the main results, Sections 3 and 4 provide some lemmas and the details of the proofs, respectively. Throughout the sequel, *C* represents a positive constant although its value may change from one place to the next, $a_{n}=O(b_{n})$ means $\vert a_{n}/{b_{n}}\vert \leq C$ and $I\{A\}$ stands for the indicator function of the set *A*.

## Main results

### Theorem 2.1

*Let* *l* *be a function slowly varying at infinity*. *Suppose that* $\{ a_{i},-\infty< i<\infty\}$ *is an absolutely summable sequence of real numbers*. *Let* $\{g(n), n\geq1\}$ *and* $\{f(n), n\geq1\}$ *be two sequences of positive constants such that*, *for some* $r\geq\max\{2,p\}$, $p\geq1$,

1. $f(n)\uparrow\infty$, $\frac{n}{f^{p}(n)}\to0$;
2. $\sum_{m=1}^{k}\log(\frac{f(m+1)}{f(m)})\sum_{n=1}^{m}\frac {ng(n)l(n)}{f(n)}=O(f^{p-1}(k)l(k))$;
3. $\sum_{m=k}^{\infty}[f^{1-r}(m+1)-f^{1-r}(m)]\sum_{n=1}^{m}\frac {ng(n)l(n)}{f(n)}=O(f^{p-r}(k)l(k))$;
4. $\sum_{m=1}^{k}[f(m+1)-f(m)]\sum_{n=1}^{m}\frac {ng(n)l(n)}{f(n)}=O(f^{p}(k)l(k))$;
5. $\sum_{m=1}^{\infty}[f^{1-r}(m+1)-f^{1-r}(m)]f^{t}(m+1)\sum_{n=1}^{m}\frac{n^{r/2}g(n)l(n)}{f(n)}<\infty$, *where* $t=\max\{0,2-p\}r/2$;
6. $\sum_{m=1}^{\infty}[f(m+1)-f(m)]f^{t'}(m+1)\sum_{n=1}^{m}\frac {n^{r/2}g(n)l(n)}{f(n)}<\infty$, *where* $t'=-\min\{2,p\}r/2$.

*Assume that* $\{X_{n}=\sum_{i=-\infty}^{\infty} a_{i}Y_{i+n}, n\geq1\}$ *is a moving average process generated by a sequence of random variables* $\{ Y_{i},-\infty< i<\infty\}$ *with mean zeros and satisfying a weak dominating condition with a dominating random variable* *Y* *and* $E\vert Y\vert ^{p}(1\vee l(f^{-1}(\vert Y\vert )))<\infty$, *where* $f^{-1}$ *is the inverse function of* *f*.

*Assume that the Rosenthal type maximal inequality of* $Y_{xj}=-xI\{Y_{j}<-x\}+Y_{j}I\{\vert Y_{j}\vert \leq x\}+xI\{Y_{j}>x\}$ *holds for the above* *r* *and all* $x> 0$. *Then*, *for all* $\varepsilon>0$,

2.1 $$\begin{aligned} \sum_{n=1}^{\infty} \frac{g(n)l(n)}{f(n)}E\Biggl\{ \max_{1\leq k\leq n} \Biggl\vert \sum _{j=1}^{k}X_{j}\Biggr\vert -\varepsilon f(n)\Biggr\} ^{+} < \infty. \end{aligned}$$

### Corollary 2.2

*If we replace conditions* (C2)-(C6) *by the following*:

(C7) $\sum_{n=1}^{k} \frac{ng(n)l(n)}{f(n)}=O(f^{p-1}(k)l(k))$, $\sum_{n=1}^{\infty}\frac{n^{r/2}g(n)l(n)}{f^{\min\{2,p\}r}(n)}<\infty$, $\sum_{n=k}^{\infty}\frac{ng(n)l(n)}{f^{r}(n)}=O(f^{p-r}(k)l(k))$.

*The other assumptions of Theorem* 2.1 *also hold*, *then*, *for all* $\varepsilon>0$, *we have*

2.2 $$\begin{aligned} \sum_{n=1}^{\infty}{g(n)l(n)}P \Biggl\{ \max_{1\leq k\leq n} \Biggl\vert \sum _{j=1}^{k}X_{j}\Biggr\vert >\varepsilon f(n)\Biggr\} < \infty. \end{aligned}$$

Conditions (C1)-(C7) can be satisfied by many sequences, we list some as the following remarks.

### Remark 2.3

Let $g(n)=n^{p\alpha-2}$, $f(n)=n^{\alpha}$ for $p\alpha>1$, and $1/2<\alpha\leq1$, assume that (1.1) holds true for $\{Y_{xj}\}$ and

$$ \textstyle\begin{cases} r>2, & 1< p\leq2,\\ r>\frac{2(p\alpha-1)}{2\alpha-1}, & p>2, \end{cases} $$

then conditions (C1)-(C7) can be verified easily by Lemma 3.1, therefore we know

2.3 $$\begin{aligned} &\sum_{n=1}^{\infty}n^{p\alpha-\alpha-2}l(n)E \Biggl\{ \max_{1\leq k\leq n} \Biggl\vert \sum _{j=1}^{k}X_{j}\Biggr\vert -\varepsilon n^{\alpha}\Biggr\} ^{+} < \infty, \end{aligned}$$

2.4 $$\begin{aligned} &\sum_{n=1}^{\infty}n^{p\alpha-2}l(n)P \Biggl\{ \max_{1\leq k\leq n} \Biggl\vert \sum _{j=1}^{k}X_{j}\Biggr\vert >\varepsilon n^{\alpha}\Biggr\} < \infty. \end{aligned}$$

Obviously, Theorem 3.1 and Corollary 3.2 from Ko [1] are the same as (2.3) and (2.4), respectively, so we extend the known results. If we take $a_{0}=1$, $a_{i}=0,i\neq0$, $l(x)=1$, let $\{Y,Y_{i},-\infty < i<\infty\}$ be a sequence of i.i.d. random variables, then $\sum_{n=1}^{\infty}n^{p\alpha-1}P\{\vert Y\vert >n^{\alpha}\}<\infty$ is equivalent to $E\vert Y\vert ^{p}<\infty$, which implies (2.4), so we can obtain Remark 1.1 from Chen [13].

### Remark 2.4

If we take $g(n)=n^{s-2}$, $f(n)=n^{s/p}$ for $s>p>1$, suppose that (1.1) holds true for $\{Y_{xj}\}$ and

$$ \textstyle\begin{cases} r>2, & 1< p\leq2,\\ r> \frac{2(s-1)p}{2s-p}, & p>2, \end{cases} $$

then conditions (C1)-(C7) can be verified easily by Lemma 3.1, so we can obtain

$$\begin{aligned} &\sum_{n=1}^{\infty}n^{s-s/p-2}l(n)E\Biggl\{ \max_{1\leq k\leq n} \Biggl\vert \sum_{j=1}^{k}X_{j} \Biggr\vert -\varepsilon n^{s/p}\Biggr\} ^{+} < \infty, \\ &\sum_{n=1}^{\infty}n^{s-2}l(n)P\Biggl\{ \max_{1\leq k\leq n} \Biggl\vert \sum_{j=1}^{k}X_{j} \Biggr\vert >\varepsilon n^{s/p}\Biggr\} < \infty. \end{aligned}$$

### Remark 2.5

If we set $g(n)=\frac{\log n}{n}$, $f(n)=(n\log n)^{1/p}$ for $1< p\leq 2$, assume that (1.1) holds true for $\{Y_{xj}\}$ and $r>4$, it is easy to see that conditions (C1)-(C7) can be satisfied by Lemma 3.1, so we can obtain

$$\begin{aligned} & \sum_{n=1}^{\infty} \frac{(\log n)^{1-1/p}l(n)}{n^{1+1/p}}E\Biggl\{ \max_{1\leq k\leq n} \Biggl\vert \sum _{j=1}^{k}X_{j}\Biggr\vert - \varepsilon(n\log n)^{1/p}\Biggr\} ^{+} < \infty, \\ & \sum_{n=1}^{\infty} \frac{(\log n)l(n)}{n}P\Biggl\{ \max_{1\leq k\leq n} \Biggl\vert \sum _{j=1}^{k}X_{j}\Biggr\vert > \varepsilon(n\log n)^{1/p}\Biggr\} < \infty. \end{aligned}$$

### Remark 2.6

Denote $g(n)=\frac{1}{n\log n}$, $f(n)=(n\log\log n)^{1/p}$ for $1< p\leq2$, assuming that (1.1) holds true for $\{Y_{xj}\}$ and $r>2$, it is easy to prove that conditions (C1)-(C7) can be satisfied by Lemma 3.1, hence the following hold:

$$\begin{aligned} &\sum_{n=1}^{\infty} \frac{l(n)}{n^{1+1/p}(\log n)(\log\log n)^{1/p}}E\Biggl\{ \max_{1\leq k\leq n} \Biggl\vert \sum _{j=1}^{k}X_{j}\Biggr\vert - \varepsilon(n\log\log n)^{1/p}\Biggr\} ^{+} < \infty, \\ &\sum_{n=1}^{\infty} \frac{l(n)}{n\log n}P\Biggl\{ \max_{1\leq k\leq n} \Biggl\vert \sum _{j=1}^{k}X_{j}\Biggr\vert > \varepsilon(n\log\log n)^{1/p}\Biggr\} < \infty. \end{aligned}$$

### Theorem 2.7

*Let* *l* *be a function slowly varying at infinity*. *Suppose that* $\{X_{n}= \sum_{i=-\infty}^{\infty} a_{i}Y_{i+n}, n\geq1\}$ *is a moving average process generated by a sequence of random variables* $\{ Y_{i},-\infty< i<\infty\}$ *with mean zeros*, *where* $\{a_{i},-\infty< i<\infty\}$ *is an absolutely summable sequence of real numbers*. *Let* $\{g(n), n\geq1\}$ *and* $\{f(n), n\geq1\}$ *be two sequences of positive constants with* $f(n)\uparrow\infty$ *and* $\{\Psi_{n}(t),n\geq1\}$ *ba a sequence of even and nonnegative functions such that*, *for each* $n\geq1$, $\Psi_{n}(t)>0$ *as* $t>0$. *Assume that*

2.5 $$\begin{aligned} \frac{\Psi_{n}(\vert t\vert )}{\vert t\vert ^{p}}\uparrow,\qquad \frac{\Psi _{n}(\vert t\vert )}{\vert t\vert ^{q}}\downarrow,\quad \textit{as } \vert t\vert \uparrow \end{aligned}$$

*for some* $1\leq p< q\leq2$, *and*

2.6 $$\begin{aligned} \sum_{n=1}^{\infty}g(n)l(n)\sum _{i=j+1}^{j+n}\frac{E\Psi _{i}(Y_{i})}{\Psi_{i}(f(n))}< \infty, \qquad\sum _{i=j+1}^{j+n}\frac{E\Psi _{i}(Y_{i})}{\Psi_{i}(f(n))}\to0,\quad \textit{as }n\to \infty \end{aligned}$$

*for any* $j\geq0$. *Assume that the Rosenthal type maximal inequality of* $Y_{nj}=Y_{j}I\{\vert Y_{j}\vert \leq f(n)\}$ *holds true for* $r=2$. *Then*, *for all* $\varepsilon>0$,

2.7 $$\begin{aligned} \sum_{n=1}^{\infty}g(n)l(n)P \Biggl\{ \max_{1\leq k\leq n} \Biggl\vert \sum _{j=1}^{k}X_{j}\Biggr\vert >\varepsilon f(n)\Biggr\} < \infty. \end{aligned}$$

## Preliminary lemmas

In order to prove the main results, we shall need the following lemmas.

### Lemma 3.1

Zhou [3]

*If* *l* *is slowly varying at infinity*, *then*

1. $\sum_{n=1}^{m}n^{s}l(n)\leq C m^{s+1}l(m)$ *for* $s>-1$ *and positive integer* *m*,
2. $\sum_{n=m}^{\infty}n^{s}l(n)\leq C m^{s+1}l(m)$ *for* $s<-1$ *and positive integer* *m*.

### Lemma 3.2

Gut [14]

*Let* $\{Y_{n}, n\geq1\}$ *be a sequence of random variables satisfy a weak dominating condition with a dominating random variable* *Y*. *For any* $b>0$, *set*

$$\begin{aligned} &Y_{i}^{\prime}=Y_{i}I\bigl\{ \vert Y_{i} \vert \leq b\bigr\} ,\qquad Y_{i}^{\prime\prime}=Y_{i}I\bigl\{ \vert Y_{i}\vert > b\bigr\} ,\\ & Y^{\prime}=YI\bigl\{ \vert Y \vert \leq b\bigr\} ,\qquad Y^{\prime\prime}=YI\bigl\{ \vert Y\vert >b\bigr\} . \end{aligned}$$

*Then for any* $a>0$ *and some constant* *C*

1. *if* $E\vert Y\vert ^{a}<\infty$, *then* $n^{-1}\sum_{i=1}^{n}E\vert Y_{i}\vert ^{a}\leq CE\vert Y\vert ^{a}$;
2. $n^{-1}\sum_{i=1}^{n}E\vert Y_{i}^{\prime} \vert ^{a}\leq C(E\vert Y^{\prime} \vert ^{a}+b^{a}P\{\vert Y\vert >b\})$;
3. $n^{-1}\sum_{i=1}^{n}E\vert Y_{i}^{\prime\prime} \vert ^{a}\leq CE\vert Y^{\prime\prime} \vert ^{a}$.

## Proofs

### Proof of Theorem 2.1

Obviously that $\sum_{k=1}^{n}X_{k}=\sum_{i=-\infty}^{\infty}a_{i}\sum_{j=i+1}^{i+n}Y_{j}$. Noting that $\sum_{i=-\infty}^{\infty} \vert a_{i}\vert <\infty$, $EY_{i}=0$, $E\vert Y\vert ^{p}(1\vee l(f^{-1}(\vert Y\vert )))<\infty$, then by Lemma 3.2 and condition (C1), for any $x>f(n)$, we conclude

$$\begin{aligned} &x^{-1}\Biggl\vert E\sum_{i=-\infty}^{\infty}a_{i} \sum_{j=i+1}^{i+n}Y_{xj}\Biggr\vert \\ &\quad = x^{-1}\Biggl\vert E\sum_{i=-\infty}^{\infty}a_{i} \sum_{j=i+1}^{i+n}(Y_{j}-Y_{xj}) \Biggr\vert \\ &\quad\leq C x^{-1}\sum_{i=-\infty}^{\infty} \vert a_{i}\vert \sum_{j=i+1}^{i+n}E \vert Y_{j}\vert I\bigl\{ \vert Y_{j}\vert > x\bigr\} \leq Cx^{-1} nE\vert Y\vert I\bigl\{ \vert Y\vert > x\bigr\} \\ &\quad\leq Cnx^{-p}E\vert Y\vert ^{p}I\bigl\{ \vert Y \vert > x\bigr\} \leq C \frac{n}{f^{p}(n)} E\vert Y\vert ^{p}I \bigl\{ \vert Y\vert > x\bigr\} \to 0, \quad \mbox{as } x\to\infty. \end{aligned}$$

Therefore, one can get

$$\begin{aligned} x^{-1}\Biggl\vert E\sum_{i=-\infty}^{\infty}a_{i} \sum_{j=i+1}^{i+n}Y_{xj}\Biggr\vert < \varepsilon/4, \end{aligned}$$

for any $\varepsilon>0$ and $x>f(n)$ large enough. Hence it follows that

4.1 $$\begin{aligned} &\sum_{n=1}^{\infty} \frac{g(n)l(n)}{f(n)} E\Biggl\{ \max_{1\leq k\leq n}\Biggl\vert \sum _{j=1}^{k}X_{j}\Biggr\vert -\varepsilon f(n)\Biggr\} ^{+} \\ &\quad\leq \sum_{n=1}^{\infty} \frac{g(n)l(n)}{f(n)} \int _{\varepsilon f(n)}^{\infty} P\Biggl\{ \max_{1\leq k\leq n} \Biggl\vert \sum_{j=1}^{k}X_{j} \Biggr\vert \geq x\Biggr\} \,dx \\ &\quad\leq C\sum_{n=1}^{\infty} \frac{g(n)l(n)}{f(n)} \int _{f(n)}^{\infty} P\Biggl\{ \max_{1\leq k\leq n} \Biggl\vert \sum_{j=1}^{k}X_{j} \Biggr\vert \geq\varepsilon x\Biggr\} \,dx \\ &\quad\leq C\sum_{n=1}^{\infty} \frac{g(n)l(n)}{f(n)} \int _{f(n)}^{\infty} P\Biggl\{ \max_{1\leq k\leq n} \Biggl\vert \sum_{i=-\infty}^{\infty}a_{i} \sum_{j=i+1}^{i+k}(Y_{j}-Y_{xj}) \Biggr\vert \geq \varepsilon x/2\Biggr\} \,dx \\ &\qquad{}+C\sum_{n=1}^{\infty} \frac{g(n)l(n)}{f(n)} \int _{f(n)}^{\infty} P\Biggl\{ \max_{1\leq k\leq n} \Biggl\vert \sum_{i=-\infty}^{\infty}a_{i} \sum_{j=i+1}^{i+k}(Y_{xj}-EY_{xj}) \Biggr\vert \geq \varepsilon x/4\Biggr\} \,dx \\ &\quad =:I_{1}+I_{2}. \end{aligned}$$

Now we want to estimate $I_{1}<\infty$. It is obvious that $\vert Y_{j}-Y_{xj}\vert \leq \vert Y_{j}\vert I\{\vert Y_{j}\vert > x\}$, then it follows by Markov’s inequality, Lemma 3.2 and conditions (C1) and (C2) that

4.2 $$\begin{aligned} I_{1}&\leq C \sum _{n=1}^{\infty}\frac{g(n)l(n)}{f(n)} \int _{f(n)}^{\infty} x^{-1}E\max _{1\leq k\leq n} \Biggl\vert \sum_{i=-\infty}^{\infty}a_{i} \sum_{j=i+1}^{i+k}(Y_{j}-Y_{xj}) \Biggr\vert \,dx \\ &\leq C\sum_{n=1}^{\infty} \frac{g(n)l(n)}{f(n)} \int _{f(n)}^{\infty} x^{-1}\sum _{i=-\infty}^{\infty} \vert a_{i}\vert \sum _{j=i+1}^{i+n}E\vert Y_{j}-Y_{xj} \vert \,dx \\ &\leq C \sum_{n=1}^{\infty} \frac{ng(n)l(n)}{f(n)} \int _{f(n)}^{\infty} x^{-1}E\vert Y\vert I \bigl\{ \vert Y\vert > x\bigr\} \,dx \\ &= C \sum_{n=1}^{\infty} \frac{ng(n)l(n)}{f(n)}\sum_{m=n}^{\infty} \int _{f(m)}^{f(m+1)} x^{-1}E\vert Y\vert I \bigl\{ \vert Y\vert > x\bigr\} \,dx \\ &\leq C \sum_{n=1}^{\infty} \frac{ng(n)l(n)}{f(n)}\sum_{m=n}^{\infty} \log \frac{f(m+1)}{f(m)}E\vert Y\vert I\bigl\{ \vert Y\vert > f(m)\bigr\} \\ &= C\sum_{m=1}^{\infty} \log \frac{f(m+1)}{f(m)}E\vert Y\vert I\bigl\{ \vert Y\vert > f(m)\bigr\} \sum _{n=1}^{m}\frac{ng(n)l(n)}{f(n)} \\ &= C\sum_{m=1}^{\infty} \Biggl[\log \frac{f(m+1)}{f(m)}\sum_{n=1}^{m} \frac{ng(n)l(n)}{f(n)}\Biggr] \sum_{k=m}^{\infty}E \vert Y\vert I\bigl\{ f(k)< \vert Y\vert \leq f(k+1)\bigr\} \\ &= C\sum_{k=1}^{\infty}E \vert Y \vert I\bigl\{ f(k)< \vert Y\vert \leq f(k+1)\bigr\} \sum _{m=1}^{k} \Biggl[\log\frac{f(m+1)}{f(m)}\sum _{n=1}^{m}\frac {ng(n)l(n)}{f(n)}\Biggr] \\ &\leq C\sum_{k=1}^{\infty}f^{p-1}(k)l(k)E \vert Y\vert I\bigl\{ f(k)< \vert Y\vert \leq f(k+1)\bigr\} \\ &\leq CE\vert Y\vert ^{p}l\bigl(f^{-1}\bigl(\vert Y \vert \bigr)\bigr)< \infty. \end{aligned}$$

Hence it remains to show that $I_{2}<\infty$. By Markov’s inequality, the Hölder inequality and the Rosenthal type maximal inequality, for $r>\max\{ 2,p\}$, it is easy to see that

4.3 $$\begin{aligned} I_{2} \leq{}& C\sum _{n=1}^{\infty}\frac{g(n)l(n)}{f(n)} \int_{f(n)}^{\infty} x^{-r}E\max _{1\leq k\leq n} \Biggl\vert \sum_{i=-\infty}^{\infty}a_{i} \sum_{j=i+1}^{i+k}(Y_{xj}-EY_{xj}) \Biggr\vert ^{r} \,dx \\ \leq{}& C\sum_{n=1}^{\infty} \frac{g(n)l(n)}{f(n)} \int _{f(n)}^{\infty} x^{-r} E \Biggl[\sum _{i=-\infty}^{\infty}\bigl(\vert a_{i} \vert ^{\frac{r-1}{r}}\bigr) \Biggl(\vert a_{i}\vert ^{1/r} \max_{1\leq k\leq n}\Biggl\vert \sum _{j=i+1}^{i+k}(Y_{xj}-EY_{xj})\Biggr\vert \Biggr) \Biggr]^{r}\,dx \\ \leq{}& C\sum_{n=1}^{\infty} \frac{g(n)l(n)}{f(n)} \int _{f(n)}^{\infty} x^{-r} \Biggl(\sum _{i=-\infty}^{\infty} \vert a_{i}\vert \Biggr)^{r-1} \Biggl(\sum_{i=-\infty}^{\infty} \vert a_{i}\vert E\max_{1\leq k\leq n}\Biggl\vert \sum _{j=i+1}^{i+k}(Y_{xj}-EY_{xj}) \Biggr\vert ^{r} \Biggr)\,dx \\ \leq{}& C\sum_{n=1}^{\infty} \frac{g(n)l(n)}{f(n)} \int _{f(n)}^{\infty} x^{-r} \sum _{i=-\infty}^{\infty} \vert a_{i}\vert \sum _{j=i+1}^{i+n}E\vert Y_{xj}-EY_{xj} \vert ^{r}\,dx \\ &{}+C\sum_{n=1}^{\infty} \frac{g(n)l(n)}{f(n)} \int _{f(n)}^{\infty} x^{-r} \sum _{i=-\infty}^{\infty} \vert a_{i}\vert \Biggl(\sum _{j=i+1}^{i+n}E\vert Y_{xj}-EY_{xj} \vert ^{2} \Biggr)^{r/2}\,dx \\ = :{}&I_{21}+I_{22}. \end{aligned}$$

For $I_{21}$, it follows by $C_{r}$ inequality, Lemma 3.2 and conditions (C1), (C3), and (C4) that

4.4 $$\begin{aligned} I_{21} \leq{}& C\sum _{n=1}^{\infty}\frac{g(n)l(n)}{f(n)} \int_{f(n)}^{\infty} x^{-r} \sum _{i=-\infty}^{\infty} \vert a_{i}\vert \sum _{j=i+1}^{i+n}\bigl[E\vert Y_{j} \vert ^{r}I\bigl\{ \vert Y_{j}\vert \leq x\bigr\} +x^{r}P\bigl(\vert Y_{j}\vert >x\bigr)\bigr]\,dx \\ \leq{}& C\sum_{n=1}^{\infty} \frac{ng(n)l(n)}{f(n)} \int _{f(n)}^{\infty} x^{-r} \bigl[E\vert Y \vert ^{r}I\bigl\{ \vert Y\vert \leq x\bigr\} +x^{r}P \bigl(\vert Y\vert >x\bigr)\bigr]\,dx \\ ={}& C\sum_{n=1}^{\infty} \frac{ng(n)l(n)}{f(n)} \sum_{m=n}^{\infty} \int_{f(m)}^{f(m+1)} \bigl[x^{-r}E\vert Y \vert ^{r}I\bigl\{ \vert Y\vert \leq x\bigr\} +P\bigl(\vert Y \vert >x\bigr)\bigr]\,dx \\ \leq{}& C\sum_{n=1}^{\infty} \frac{ng(n)l(n)}{f(n)} \sum_{m=n}^{\infty} \bigl[f^{1-r}(m+1)-f^{1-r}(m)\bigr]E\vert Y\vert ^{r}I\bigl\{ \vert Y\vert \leq f(m+1)\bigr\} \\ &{ }+C\sum_{n=1}^{\infty} \frac{ng(n)l(n)}{f(n)} \sum_{m=n}^{\infty} \bigl[f(m+1)-f(m)\bigr]P\bigl(\vert Y\vert >f(m)\bigr) \\ ={}&C\sum_{m=1}^{\infty} \bigl[f^{1-r}(m+1)-f^{1-r}(m)\bigr]E\vert Y\vert ^{r}I\bigl\{ \vert Y\vert \leq f(m+1)\bigr\} \sum _{n=1}^{m}\frac{ng(n)l(n)}{f(n)} \\ &{}+C\sum_{m=1}^{\infty} \bigl[f(m+1)-f(m)\bigr]P\bigl(\vert Y\vert >f(m)\bigr)\sum _{n=1}^{m}\frac{ng(n)l(n)}{f(n)} \\ ={}&C\sum_{m=1}^{\infty} \Biggl\{ \bigl[f^{1-r}(m+1)-f^{1-r}(m)\bigr]\sum _{n=1}^{m}\frac{ng(n)l(n)}{f(n)} \Biggr\} \sum _{k=1}^{m}E\vert Y\vert ^{r}I\bigl\{ f(k)< \vert Y\vert \leq f(k+1)\bigr\} \\ &{}+C\sum_{m=1}^{\infty} \Biggl\{ \bigl[f(m+1)-f(m)\bigr]\sum_{n=1}^{m} \frac{ng(n)l(n)}{f(n)} \Biggr\} \sum_{k=m}^{\infty}P \bigl\{ f(k)< \vert Y\vert \leq f(k+1)\bigr\} \\ ={}&C\sum_{k=1}^{\infty}E \vert Y \vert ^{r}I\bigl\{ f(k)< \vert Y\vert \leq f(k+1)\bigr\} \sum _{m=k}^{\infty} \Biggl\{ \bigl[f^{1-r}(m+1)-f^{1-r}(m) \bigr]\sum_{n=1}^{m}\frac {ng(n)l(n)}{f(n)} \Biggr\} \\ &{}+C\sum_{k=1}^{\infty}P\bigl\{ f(k)< \vert Y\vert \leq f(k+1)\bigr\} \sum_{m=1}^{k} \Biggl\{ \bigl[f(m+1)-f(m)\bigr]\sum_{n=1}^{m} \frac {ng(n)l(n)}{f(n)} \Biggr\} \\ \leq{}& C\sum_{k=1}^{\infty} f^{p-r}(k)l(k) E\vert Y\vert ^{r}I\bigl\{ f(k)< \vert Y \vert \leq f(k+1)\bigr\} \\ &{}+C\sum_{k=1}^{\infty}f^{p}(k)l(k)P \bigl\{ f(k)< \vert Y\vert \leq f(k+1)\bigr\} \\ \leq{}& CE\vert Y\vert ^{p}l\bigl(f^{-1}\bigl(\vert Y \vert \bigr)\bigr)< \infty. \end{aligned}$$

Finally we want to show that $I_{22}<\infty$, by $C_{r}$ inequality, Lemma 3.2 and conditions (C1), (C5), and (C6), it follows that

4.5 $$\begin{aligned} I_{22} \leq{}& C\sum _{n=1}^{\infty}\frac{n^{r/2}g(n)l(n)}{f(n)} \int_{f(n)}^{\infty } x^{-r} \bigl[\bigl(E\vert Y\vert ^{2}I\bigl\{ \vert Y\vert \leq x\bigr\} \bigr)^{r/2}+x^{r}P^{r/2}\bigl(\vert Y\vert >x \bigr)\bigr]\,dx \\ \leq{}& C\sum_{n=1}^{\infty} \frac{n^{r/2}g(n)l(n)}{f(n)} \sum_{m=n}^{\infty} \int_{f(m)}^{f(m+1)} \bigl[x^{-r}\bigl(E\vert Y \vert ^{2}I\bigl\{ \vert Y\vert \leq x\bigr\} \bigr)^{r/2}+P^{r/2} \bigl(\vert Y\vert >x\bigr)\bigr]\,dx \\ \leq{}& C\sum_{n=1}^{\infty} \frac{n^{r/2}g(n)l(n)}{f(n)} \sum_{m=n}^{\infty} \bigl[f^{1-r}(m+1)-f^{1-r}(m)\bigr]\bigl(E\vert Y\vert ^{2}I\bigl\{ \vert Y\vert \leq f(m+1)\bigr\} \bigr)^{r/2} \\ &{}+C\sum_{n=1}^{\infty} \frac{n^{r/2}g(n)l(n)}{f(n)} \sum_{m=n}^{\infty} \bigl[f(m+1)-f(m)\bigr]P^{r/2}\bigl(\vert Y\vert >f(m)\bigr) \\ ={}&C\sum_{m=1}^{\infty} \bigl[f^{1-r}(m+1)-f^{1-r}(m)\bigr]\bigl(E\vert Y\vert ^{2}I\bigl\{ \vert Y\vert \leq f(m+1)\bigr\} \bigr)^{r/2} \sum_{n=1}^{m}\frac{n^{r/2}g(n)l(n)}{f(n)} \\ &{}+ \sum_{m=1}^{\infty} \bigl[f(m+1)-f(m)\bigr]P^{r/2}\bigl(\vert Y\vert >f(m)\bigr)\sum _{n=1}^{m}\frac{n^{r/2}g(n)l(n)}{f(n)} \\ \leq{}& C\sum_{m=1}^{\infty} \Biggl\{ \bigl[f^{1-r}(m+1)-f^{1-r}(m)\bigr]\sum _{n=1}^{m}\frac{n^{r/2}g(n)l(n)}{f(n)} f^{\max\{0,2-p\}r/2}(m+1) \Biggr\} \\ &{}\times \bigl(E\vert Y\vert ^{\min\{p,2\}}\bigr)^{r/2} \\ &{}+C\sum_{m=1}^{\infty} \Biggl\{ \bigl[f(m+1)-f(m)\bigr]\sum_{n=1}^{m} \frac{n^{r/2}g(n)l(n)}{f(n)}f^{-\min\{2,p\}r/2}(m) \Biggr\} \bigl(E\vert Y\vert ^{\min\{p,2\}}\bigr)^{r/2} \\ < {}& \infty. \end{aligned}$$

Hence the proof of (2.1) is completed by combining (4.1)-(4.5). □

### Proof of Theorem 2.7

Clearly $\sum_{j=1}^{k}X_{j}=\sum_{i=-\infty}^{\infty}a_{i}\sum_{j=i+1}^{i+k}Y_{j}$. Noting that $\sum_{i=-\infty}^{\infty} \vert a_{i}\vert <\infty$ and $EY_{j}=0$, then by (2.5) and (2.6), we know

$$\begin{aligned} &\frac{1}{f(n)}\max_{1\leq k\leq n}\Biggl\vert E\sum _{i=-\infty}^{\infty }a_{i}\sum _{j=i+1}^{i+k}Y _{nj}\Biggr\vert \\ &\quad \leq \frac{1}{f(n)}\sum_{i=-\infty}^{\infty} \vert a_{i}\vert \sum_{j=i+1}^{i+n}E \vert Y_{j}\vert I\bigl\{ \vert Y_{j}\vert > f(n) \bigr\} \\ &\quad\leq\frac{1}{f^{p}(n)}\sum_{i=-\infty}^{\infty} \vert a_{i}\vert \sum_{j=i+1}^{i+n}E \vert Y_{j}\vert ^{p}I\bigl\{ \vert Y_{j} \vert > f(n)\bigr\} \leq C\sum_{i=-\infty}^{\infty} \vert a_{i}\vert \sum_{j=i+1}^{i+n} E\frac{\Psi _{j}(Y_{j})}{\Psi_{j}(f(n))} \\ &\quad\to 0, \quad\mbox{as }n\to\infty. \end{aligned}$$

Hence for *n* large enough and any $\varepsilon>0$, we obtain

$$\frac{1}{f(n)}\max_{1\leq k\leq n}\Biggl\vert E\sum _{i=-\infty}^{\infty }a_{i}\sum _{j=i+1}^{i+k}Y _{nj}\Biggr\vert < \varepsilon/4. $$

Then one can get

$$\begin{aligned} &\sum_{n=1}^{\infty}g(n)l(n) P \Biggl\{ \max_{1\leq k\leq n}\Biggl\vert \sum _{j=1}^{k}X_{j}\Biggr\vert >\varepsilon f(n) \Biggr\} \\ &\quad\leq C\sum_{n=1}^{\infty}g(n)l(n) P \Biggl\{ \max_{1\leq k\leq n} \Biggl\vert \sum _{i=-\infty}^{\infty}a_{i}\sum _{j=i+1}^{i+k}(Y_{j}-Y_{nj})\Biggr\vert > \varepsilon f(n)/2 \Biggr\} \\ &\qquad{}+C\sum_{n=1}^{\infty}g(n)l(n) P \Biggl\{ \max_{1\leq k\leq n} \Biggl\vert \sum _{i=-\infty}^{\infty}a_{i}\sum _{j=i+1}^{i+k}(Y_{nj}-EY_{nj})\Biggr\vert > \varepsilon f(n)/4 \Biggr\} \\ &\quad =:J_{1}+J_{2}. \end{aligned}$$

By Markov’s inequality, (2.5), and (2.6), it is easy to check that

$$\begin{aligned} J_{1}&\leq C \sum _{n=1}^{\infty}\frac{g(n)l(n)}{f(n)} E\max _{1\leq k\leq n} \Biggl\vert \sum_{i=-\infty}^{\infty}a_{i} \sum_{j=i+1}^{i+k}(Y_{j}-Y_{nj}) \Biggr\vert \\ &\leq C\sum_{n=1}^{\infty} \frac{g(n)l(n)}{f(n)} \sum_{i=-\infty}^{\infty} \vert a_{i}\vert \sum_{j=i+1}^{i+n}E \vert Y_{j}\vert I\bigl\{ \vert Y_{j}\vert > f(n) \bigr\} \\ &\leq C\sum_{n=1}^{\infty} \frac{g(n)l(n)}{f^{p}(n)} \sum_{i=-\infty}^{\infty} \vert a_{i}\vert \sum_{j=i+1}^{i+n}E \vert Y_{j}\vert ^{p}I\bigl\{ \vert Y_{j} \vert > f(n)\bigr\} \\ &\leq C \sum_{i=-\infty}^{\infty} \vert a_{i}\vert \sum_{n=1}^{\infty}{g(n)l(n)} \sum_{j=i+1}^{i+n} E\frac{\Psi_{j}(Y_{j})}{\Psi_{j}(f(n))}< \infty. \end{aligned}$$

It follows from Markov’s inequality, the Hölder inequality, the Rosenthal type inequality, (2.5), and (2.6) that

$$\begin{aligned} J_{2} &\leq C\sum _{n=1}^{\infty}\frac{g(n)l(n)}{f^{2}(n)}E\max _{1\leq k\leq n} \Biggl\vert \sum_{i=-\infty}^{\infty}a_{i} \sum_{j=i+1}^{i+k}(Y_{nj}-EY_{nj}) \Biggr\vert ^{2} \\ &\leq C\sum_{n=1}^{\infty} \frac{g(n)l(n)}{f^{2}(n)} \sum_{i=-\infty}^{\infty} \vert a_{i}\vert \Biggl(\sum_{j=i+1}^{i+n}E \vert Y_{nj}-EY_{nj}\vert ^{2}\Biggr) \\ &\leq C\sum_{n=1}^{\infty} \frac{g(n)l(n)}{f^{2}(n)} \sum_{i=-\infty}^{\infty} \vert a_{i}\vert \Biggl(\sum_{j=i+1}^{i+n}EY^{2}_{j}I \bigl\{ \vert Y_{j}\vert \leq f(n)\bigr\} \Biggr) \\ &\leq C\sum_{n=1}^{\infty} \frac{g(n)l(n)}{f^{q}(n)} \sum_{i=-\infty}^{\infty} \vert a_{i}\vert \Biggl(\sum_{j=i+1}^{i+n}E \vert Y_{j}\vert ^{q}I\bigl\{ \vert Y_{j} \vert \leq f(n)\bigr\} \Biggr) \\ &\leq C \sum_{i=-\infty}^{\infty} \vert a_{i}\vert \sum_{n=1}^{\infty}{g(n)l(n)} \sum_{j=i+1}^{i+n} E\frac{\Psi_{j}(Y_{j})}{\Psi_{j}(f(n))}< \infty. \end{aligned}$$

Thus we have completed the proof of Theorem 2.7. □
